# Case report: ALK-positive histiocytosis presented as bilateral synchronous breast masses with long-term remission on crizotinib

**DOI:** 10.3389/fmed.2023.1288849

**Published:** 2023-11-29

**Authors:** Yuhang Zhou, Marisabel Hurtado-Castillo, Om Pandey

**Affiliations:** Department of Oncology, University of Texas at Austin, Austin, TX, United States

**Keywords:** ALK1, histiocytosis, breast, brain, crizotinib

## Abstract

ALK-positive histiocytosis (APH) is a rare type of histiocytic neoplasm with characteristic *ALK* (Anaplastic Lymphoma Kinase) gene translocation and fusion, with only 27 reported cases in the literature. In this study, we report the first case of synchronous bilateral breast involvement of ALK-positive histiocytosis on initial presentation in a 46-year-old Hispanic woman. APH was diagnosed by the confirmation of clonal histiocyte proliferation with ALK overexpression on IHC and the presence of *KIF5B-ALK* gene fusion from her breast and lung biopsies. The patient in our study is currently under complete and long-term remission with crizotinib treatment (an ALK inhibitor). This report expands on the clinical manifestation of APH, emphasizes the importance of ALK detection in histiocytic diseases, and provides the efficacy and long-term prognosis of the ALK inhibitor therapy for APH.

## 1 Introduction

ALK-positive histiocytosis (APH), a unique subtype of histiocyte neoplasms, is a recently defined clonal histiocytic neoplasm driven by *ALK* amplification and translocation with a wide range of disease presentations involving multiple organs, such as the liver, bone, lung, brain, and skin (1–7). The majority of APH cases affect young patients of Asian descent, with a varying degree of clinical severity ranging from spontaneous resolution to life-threatening complications (1, 2, 6, 8, 9). APH is diagnosed with the combination of pathologic confirmation of ALK-overexpressed histiocytic proliferation and genetic evidence of *ALK* translocation, with the fusion product *KIF5B-ALK* as the most common genetic aberration (6, 10). In this study, we report the first case of synchronous bilateral breast masses based on the initial presentation of APH in the literature. We describe the clinical, histological, radiological, and genetic findings of our patient, along with her treatment response to crizotinib.

## 2 Case presentation

### 2.1 Clinical presentation

This study describes the case report of a 46-year-old Hispanic woman with hypertension and pre-diabetes who presented to her primary care provider in June 2020 due to enlarging bilateral breast masses, left breast nipple ulceration, chronic neck pain, and new right-sided shoulder pain. The patient denied experiencing weight loss, fever, night sweats, dyspnea, cough, hemoptysis, headache, weakness, or neurologic symptoms. She had no family history of neoplasms or blood disorders, and her vital signs were within normal limits. Notably, before the onset of symptoms in 2020, she had annual mammogram screenings in 2018 and 2019, the results of which were normal.

Her initial physical examination revealed medium-sized breasts with grade III ptosis and dense breast tissue. The patient had a small ulceration on her left nipple with minimal discharge. She presented with a 1 cm palpable solid breast mass at the 2:00 position, which was tender upon palpation, in addition to a 2 cm palpable firm breast mass on the right side at 11:00 and an adjacent 1.5 cm mass at 9:00. All masses exhibited mobility on her skin and chest walls. No palpable axillary/internal mammary or clavicular lymph nodes were found upon examination. She had an intact neurological, abdominal, and musculoskeletal exam. Her blood counts and metabolic panel were within normal ranges, with the exception of a mildly elevated alkaline phosphatase at 172. Meanwhile, her LDH level remained within the normal range at 181.

**Figure 1:**
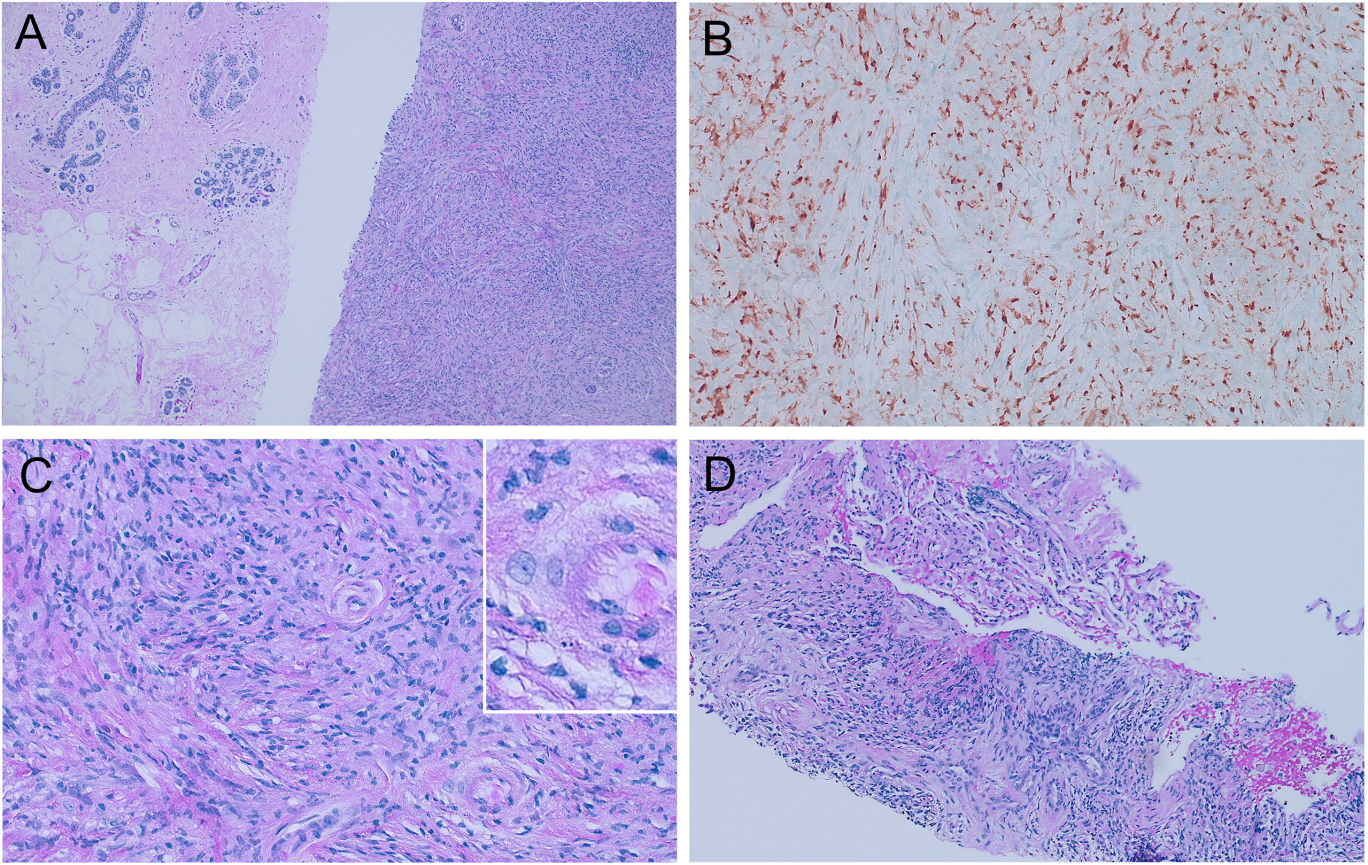
Histologic features of ALK-positive histiocytosis. **(A)** Breast biopsy with 100X hematoxylin and eosin staining showing the contrast between normal breast tissues on the left and APH on the right, which consists of sheets of packed proliferating cells with eosinophilic cytoplasm. **(B)** Immunohistochemistry staining showing positive ALK1 expression. **(C)** 400X H&E staining shows foamy histiocytes with irregularly folded or lobulated nuclei. **(D)** Lung biopsy with 100X H&E staining showed similar histologic findings of APH.

Subsequently, her diagnostic mammography in August 2020 showed bilateral BI-RADS category 4C (high suspicion for malignancy) breast masses. She underwent a fine needle biopsy of the two right breast masses and a punch biopsy of the left side nipple ulceration, of which all three samples were consistent with clonal expansion of histiocytic neoplasms. Immunohistochemistry stains (IHC) were positive for ALK1, CD163, CD68, and S100 and negative for HMWK, cytokeratin, p63, CD34, desmin, actin, STAT6, SOX10, AFB, and GMS (Figure 1). The next-generation sequencing test (FoundationOne CDx) conducted on her breast biopsy sample revealed a positive *KIF5B-ALK* fusion, a low tumor mutational burden (1 muts/Mb), stable microsatellite status, and no other driver mutations or alterations were detected (including but not limited to *BRAF, PIK3CA*, and *MAP2K1*).

**Figure 2:**
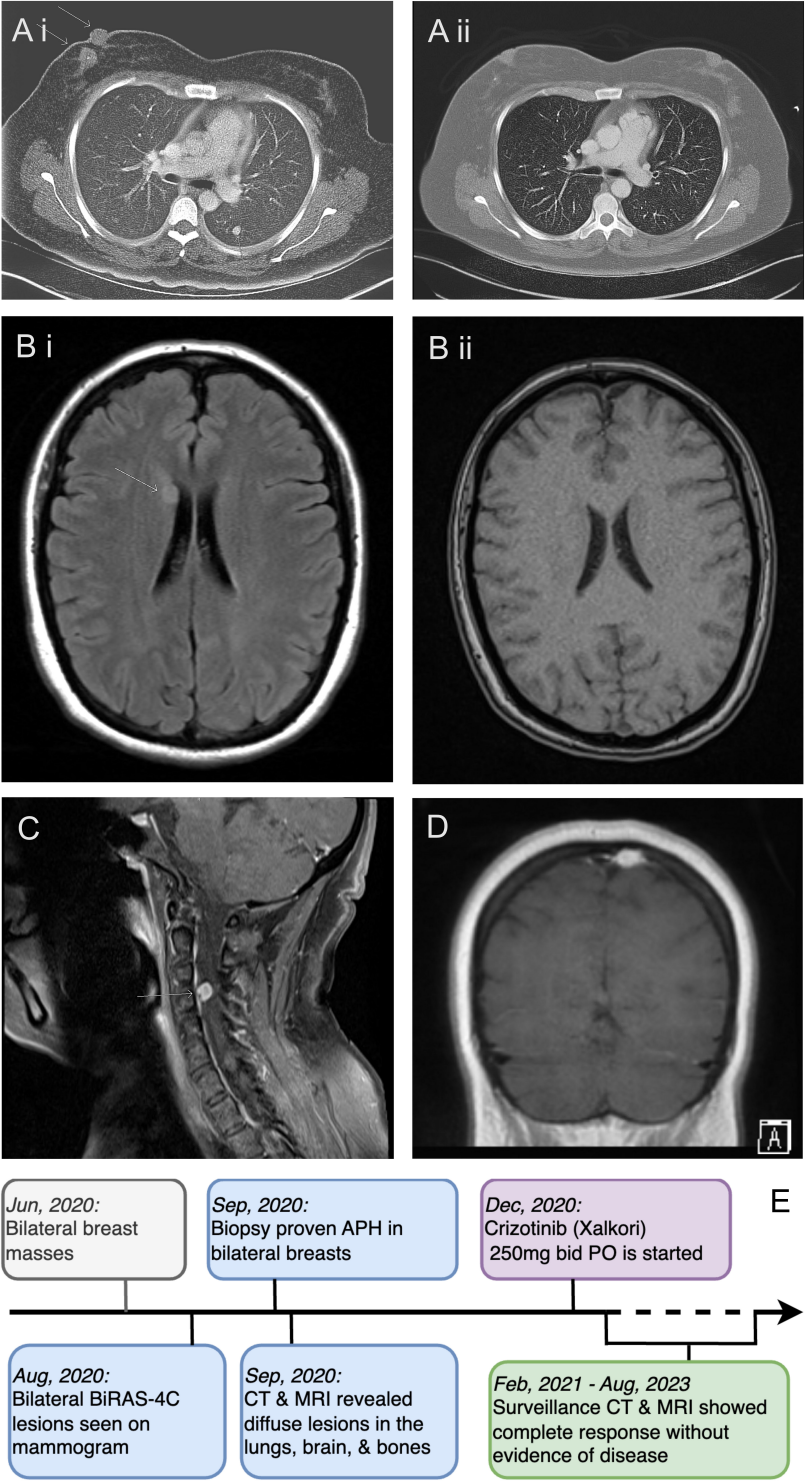
Radiologic features of APH and case timeline. **(A)** CT scan of the chest with contrast before **(A i)** and after **(A ii)** starting crizotinib. Near-complete resolution of the right-side breast mass and left lung nodule are shown. **(B)** MRI of the brain before **(B i)** and after **(B ii)** crizotinib treatment, showing a complete response of the right caudate head lesion. **(C)** MRI of the spine at initial presentation showing soft disease involvement at the right C3-4 foramen. **(D)** MRI of the brain showing the calvarial lesion in the posterior left parietal bone at the vertex before treatment. **(E)** A clinical, diagnostic, and treatment timeline.

To assess the extent of her illness, the patient underwent several diagnostic tests in September 2020. These included a brain MRI, a CT scan of the chest, abdomen, and pelvis, and an MR spectroscopy of the cervical spine. The results indicated that the patient suffered from extensive metastasis, with numerous lung nodules (the largest measuring 9 mm), multiple small brain intra-axial enhancing nodules, parietal bone lesions, multiple parotid nodules, and a dumbbell-shaped soft tissue mass at the right C3-4 foramen, without any lesions in her liver (Figure 2). A nuclear imaging bone scan discovered disease involvement in the skull, vertex sternal, right femur, and mandible. Her bone marrow biopsy result showed no evidence of the disease with age-appropriate cellularity. Her left lung nodule biopsy result showed histiocytic proliferation, with similar IHC patterns as her breast masses. According to the clinical presentations, histology, immunophenotyping, and genetic workup, this case confirmed a diagnosis of ALK-positive histiocytosis (Figures 1, 2).

### 2.2 Treatment

Our patient started taking crizotinib 250 mg PO twice daily 3 months after the initial presentation in December 2020. The decision to use crizotinib over other ALK inhibitors was based on its reported efficacy, adverse effect profile, and patient preference. We also plan to reserve the newer generation of ALK inhibitors and chemotherapy for subsequent therapy at the time of progression. Surgery and radiation were not necessary in this case because of the diffuse involvement of the APH and the lack of severe space-occupying symptoms of the lesions. She tolerated crizotinib well without any short-term or long-term adverse effects. The patient had a dramatic clinical and radiological response immediately after the treatment. Her left breast ulceration quickly healed within 2 weeks. Her right-side breast masses disappeared clinically in 2 months. A follow-up CT scan 4 months after treatment initiation showed complete resolution of her lung, breast, brain, and bone diseases (Figure 2). She continued taking crizotinib with good compliance. Her most recent mammography in August 2023 was normal. She had been receiving regular CT scans every 6 months, along with periodic bone scans, brain MRIs, and clinic visits. There was no evidence of disease recurrence or treatment-related adverse effects at the time of submission. A timeline of her clinical presentation, diagnostic journey, and treatment initiation is illustrated in Figure 2E.

## 3 Discussion

**Figure 3:**
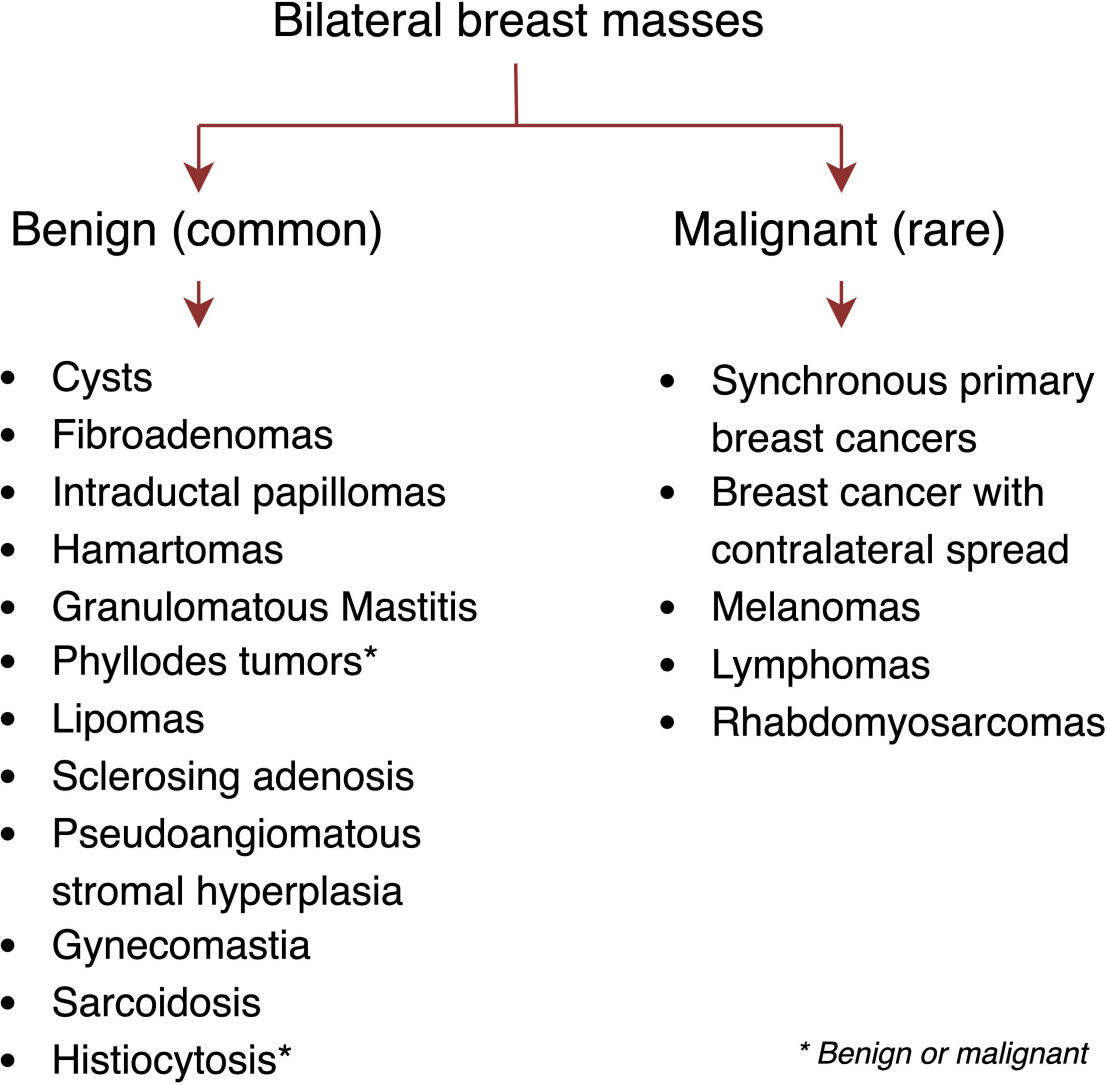
Differential diagnosis of bilateral breast masses.

Bilateral breast masses are an uncommon clinical presentation. The most common etiologies are benign findings such as breast cysts, fibroadenomas, and intraductal papillomas (a differential diagnosis summarized in Figure 3). Malignant causes include primary breast cancer, metastatic diseases from other primary sites (melanoma, lymphoma), and other breast-originated malignancies such as sarcomas. Several reports described histiocytosis presented as bilateral breast masses, including Langerhans cell histiocytosis (11), Rosai-Dorfman syndrome (12), and Erdheim-Chester disease (13). To the best of our knowledge, this case is the first report of ALK-positive histiocytosis presented as synchronous bilateral breast masses.

Histiocytes are crucial immune modulators that are part of the mononuclear phagocyte system. Monocytes are produced in the bone marrow from hematologic stem cells, circulate via blood, and eventually undergo differentiation into histiocytes, which are involved in wound healing, host defense, and regulation of inflammatory responses (8). The Histiocytic Society classifies histiocytic disorders into five groups in their 2016 guideline, in which APH is included in the L group (8). While the majority of histiocytic disorders are benign, malignant histiocytoses do exist and are characterized by anaplastic histology. In addition, they are often associated with chromosomal defects and other malignancies, such as follicular lymphoma, hairy cell leukemia, CLL, and ALL (8, 14, 15).

**Figure 4:**
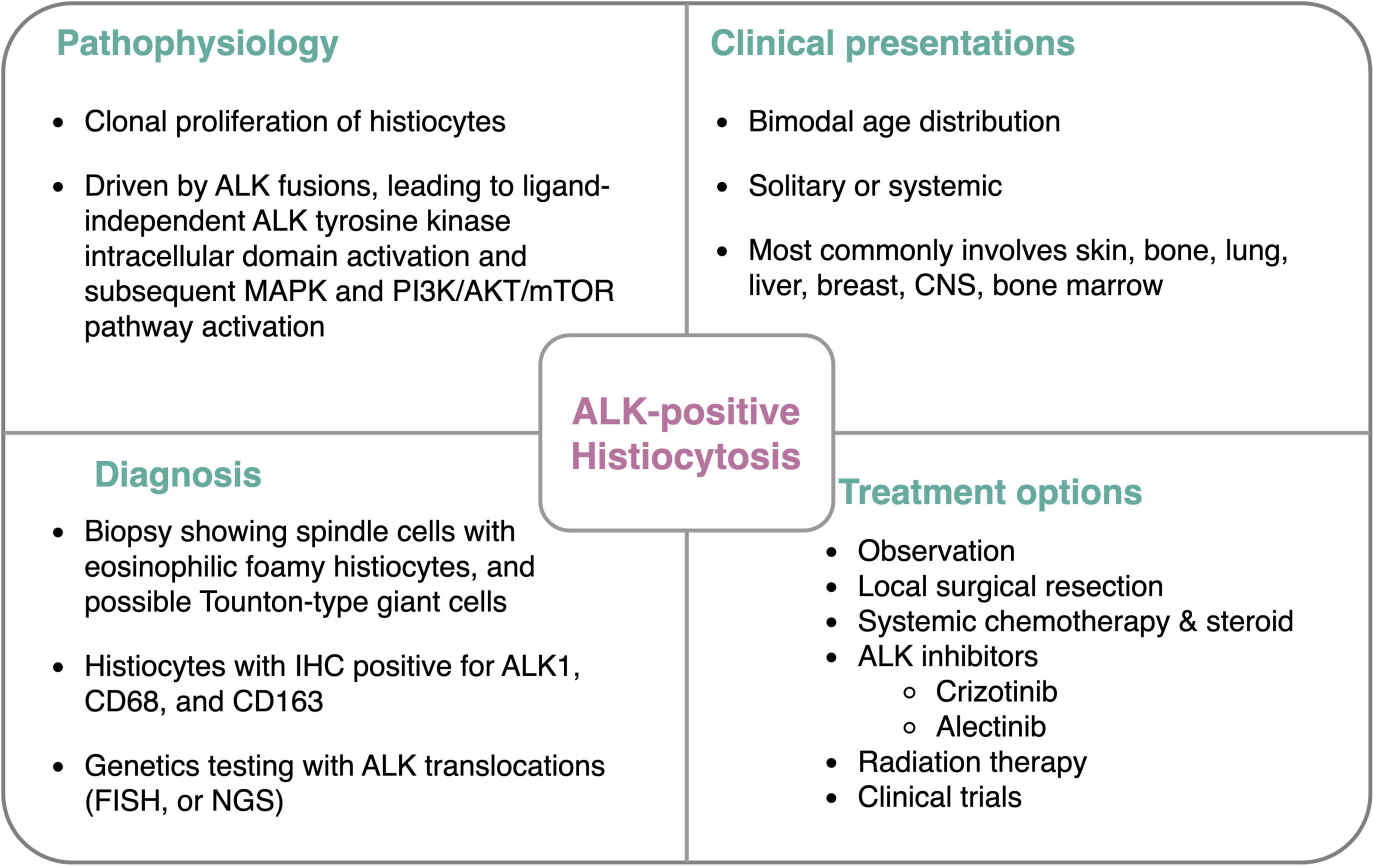
Features of ALK-positive histiocytosis.

ALK-positive histiocytosis (APH) was first reported in 2008, with three cases with characteristic ALK expression on IHC (1). Since then, <30 cases have been reported with a variety of clinical presentations and responses to different treatments. APH is driven by ALK pathway overactivation, given the fact that almost all cases of ALK have chromosomal changes that lead to *ALK* fusion and overexpression and that patients respond well to ALK inhibitors (16, 17). To the best of our knowledge, there are currently 27 reported cases of APH, including the one in this study. The age distribution favors a bimodal pattern, with 10 patients younger than 3 years of age and 17 young adult/adult patients with a median age of 32 years. The sites of the disease vary throughout the organ systems. Most of the pediatric cases involve the liver, spleen, skin, and bone marrow (1, 6, 18), while most of the adult cases demonstrate visceral diseases of the lung, bone, and central nervous system, among others (2, 3, 19). There are five cases of breast involvement with APH, which were all presented as unilateral breast lesions (20–22). No previously reported APH was found in Hispanic patients, excluding this study. In terms of cytogenetics, ALK overexpression and rearrangement were found in all APH cases. Out of the 19 *ALK* fusion-positive cases, 16 of them are positive for *KIF5B-ALK* (6, 10, 23). Other *ALK* partners include *TPM3, TRIM33*, and *EML4* (1, 24, 25). The pathophysiology, clinical presentations, diagnosis, and treatment options of APH are summarized in Figure 4.

The *ALK* gene, located on chromosome 2p23, encodes the anaplastic lymphoma kinase (ALK) receptor tyrosine kinase (CD246), which is a member of the insulin receptor superfamily. ALK is robustly expressed in the developing nervous system, with a diminished presence in adults. The physiological function of ALK has been shown in brain development and neuron differentiation (26). In adults, ALK expression is found in the brain, GI system, testis, and prostate but not in the lung or lymphatic systems (27). Upon ALK activation by ligand engagement or fusion protein activation, downstream activation of several pathways (JAK-STAT3, PI3K, mTOR, and MAPK) is achieved by phosphorylation by ALK, resulting in cellular proliferation and transformation (28).

In tumorigenesis, ALK amplifications and fusions have been linked to various hematological malignancies and tumor types, including non-small cell lung cancer, anaplastic large cell lymphomas, neuroblastoma, and rhabdomyosarcoma (29). The recent advancement and popularity of next-generation sequencing have made the detection of ALK gene fusion variants easier and therefore more frequent. The most common fusion genes in non-small cell lung cancer include but are not limited to, *ALK/EML4, ALK/KIF5B, ALK/TFG*, and *ALK/PTPN3* (27, 30).

The prognosis for APH is usually fair. Most of the APH cases were able to achieve stable disease or complete remission after systemic chemotherapy, surgery, or targeted therapy. ALK inhibitors were used in three cases (crizotinib or alectinib) with an excellent response (16, 18, 31). Syrykh et al. (9) reported a case of APH associated with CLL/SLL that achieved complete remission with ibrutinib, suggesting that BTK inhibitors may also be effective in treating APH. The long-term outcome of APH has yet to be observed. The resistance of TKI and its subsequent relapse have not been reported yet, but it is possible in the future. As reported by Zeng et al. (23), an acquired *ALK* L1196M mutation was detected 11 months after disease progression while on crizotinib treatment in a case of lung adenocarcinoma with *KIF5B-ALK* fusion. In this case, the treatment was switched to ceritinib, which was effective on the *ALK* L1196M mutation.

The treatment options for APH vary depending on the clinical presentation and severity. Some cases report spontaneous resolution of lesions without any treatment in infants. Most of the reported cases of APH underwent surgical resection of easily accessible lesions with a high cure rate. In the unresectable and metastatic APH settings, no clinical trials are currently registered, given the rarity of this disease. Single-agent and combination chemotherapy with or without local radiation have been used in some reports. Considering the pivotal role of ALK pathway activation in APH, ALK inhibition is a reasonable option. Crizotinib (brand name Xalkori) is a first-generation tyrosine kinase inhibitor targeting anaplastic lymphoma kinase (ALK), ROS1, and cMET (32–34). It is approved by the FDA for the treatment of ALK- or ROS1-positive non-small cell lung cancer and young patients with ALK-positive relapsed or refractory systemic anaplastic large cell lymphoma (FDA package insert). Crizotinib is generally well-tolerated. The common side effects include nausea, diarrhea, vomiting, edema, constipation, elevated transaminases, fatigue, low appetite, dizziness, vision disorders, and neuropathy. Serious adverse effects, which include interstitial lung disease, hepatotoxicity, QT interval prolongation, bradycardia, and vision loss, are rare. Our patient did not experience any short-term or long-term adverse effects while on crizotinib. Other ALK inhibitors include, but are not limited to, second-generation alectinib, ceritinib, brigatinib, and third-generation lorlatinib. To date, only crizotinib and alectinib have been reported to be effective in APH (16, 31, 35). It is unclear whether TKIs need to be continued for life for the treatment of APH. In non-small cell lung cancer with residual disease, cessation of TKIs almost always leads to disease progression, indicating that TKIs are likely cytostatic rather than cytotoxic (33, 36). New resistance mutations in ALK or other driver genes have been identified in patients who progress on crizotinib (37–39). In this case, we plan to continue crizotinib indefinitely until disease progression or serious side effects occur. We will consider performing a molecular analysis of the new lesions by re-biopsy.

In conclusion, we present the case report of a 46-year-old woman with ALK-positive histiocytosis of the bilateral breasts with lung, brain, and bone metastasis. She was treated successfully with an ALK inhibitor, crizotinib, with a complete clinical and radiographic response for more than 2 years. Our report expands the clinical spectrum of APH with synchronous bilateral breast masses as the initial presentation and is also the first report of APH in a Hispanic patient. Histiocytosis should be included in the differential diagnosis of bilateral breast tumors. The success of ALK inhibitors in this and other reports further emphasized the importance of testing for ALK in all histiocytic disorders. Early detection and diagnosis of APH can help avoid high-risk surgical excision or cytotoxic systemic therapy, which can prevent potential adverse effects and lead to a better quality of life for the selected patients.

## Data availability statement

The original contributions presented in the study are included in the article/supplementary material, further inquiries can be directed to the corresponding author.

## Ethics statement

Written informed consent was obtained from the individual(s) for the publication of any potentially identifiable images or data included in this article.

## Author contributions

YZ: Conceptualization, Data curation, Writing—original draft, Writing—review & editing. MH-C: Resources, Writing—review & editing. OP: Supervision, Validation, Writing—review & editing.

## References

[1] Chan JKC Lamant L Algar E Delsol G Tsang WYW Lee KC. ALK+ histiocytosis: a novel type of systemic histiocytic proliferative disorder of early infancy. Blood. (2008) 112:2965–8. 10.1182/blood-2008-03-147017 18660380

[2] Lucas CHG Gilani A Solomon DA Liang X Maher OM Chamyan G. ALK-positive histiocytosis with KIF5B-ALK fusion in the central nervous system. Acta Neuropathol. (2019) 138:335–7. 10.1007/s00401-019-02027-7 31119374 PMC6712982

[3] Jaber OI Jarrah D. Al, Hiasat M, Hussaini M Al. ALK-positive histiocytosis: a case report and literature review. Turk Patoloji Derg. (2021) 37:172–7. 10.5146/tjpath.2020.01507 33973641 PMC10512673

[4] Yuan CT Chen JS Huang YL Zhang MS Hsieh MS. ALK-positive histiocytosis presenting as a solitary pulmonary nodule. Br J Haematol. (2022) 199:7. 10.1111/bjh.18371 35867481

[5] Swain F Williams B Barbaro P. ALK-positive histiocytosis with peripheral blood histiocytes: a case report. Acta Haematol. (2021) 144:218–21. 10.1159/000508524 32721959

[6] Chang KTE Tay AZE Kuick CH Chen H Algar E Taubenheim N. ALK-positive histiocytosis: an expanded clinicopathologic spectrum and frequent presence of KIF5B-ALK fusion. Mod Pathol. (2019) 32:598–608. 10.1038/s41379-018-0168-6 30573850

[7] Liu W Liu HJ Wang WY Tang Y Zhao S Zhang WY. Multisystem ALK-positive histiocytosis: a multi-case study and literature review. Orphanet J Rare Dis. (2023) 18:53. 10.1186/s13023-023-02649-x 36915094 PMC10010018

[8] Emile JF Abla O Fraitag S Horne A Haroche J Donadieu J. Revised classification of histiocytoses and neoplasms of the macrophage-dendritic cell lineages. Blood. (2016) 127:2672–81. 10.1182/blood-2016-01-690636 26966089 PMC5161007

[9] Syrykh C Ysebaert L Péricart S Evrard SM Meggetto F Kanoun S. ALK-positive histiocytosis associated with chronic lymphocytic leukaemia/small lymphocytic lymphoma: a multitarget response under ibrutinib. Virchows Arch. (2021) 478:779–83. 10.1007/s00428-020-02937-y 33011863

[10] Guo Y Qu HB Ning G Jia FL Liu H Ma XM. Case report: ALK-Positive histiocytosis with KIF5B-ALK fusion in cerebrum-disseminated lesions in a child. Front Oncol. (2022) 12:858939. 10.3389/fonc.2022.858939 35359354 PMC8960947

[11] O'Kane D Jenkinson H Carson J. Langerhans cell histiocytosis associated with breast carcinoma successfully treated with topical imiquimod. Clin Exp Dermatol. (2009) 34:e829–32. 10.1111/j.1365-2230.2009.03569.x 19843082

[12] Green I Dorfman RF Rosai J. Breast involvement by extranodal Rosai-Dorfman disease: report of seven cases. Am J Surg Pathol. (1997) 21:664–8. 10.1097/00000478-199706000-00006 9199644

[13] Provenzano E Barter SJ Wright PA Forouhi P Allibone R Ellis IO. Erdheim-chester disease presenting as bilateral clinically malignant breast masses. Am J Surg Pathol. (2010) 34:584–8. 10.1097/PAS.0b013e3181d39a3d 20216377

[14] Bagnasco F Zimmermann SY Egeler RM Nanduri VR Cammarata B Donadieu J. Langerhans cell histiocytosis and associated malignancies: a retrospective analysis of 270 patients. Eur J Cancer. (2022) 172:138–45. 10.1016/j.ejca.2022.03.036 35772351

[15] Egeler RM Neglia JP Aricò M Favara BE Heitger A Nesbit ME. Acute leukemia in association with Langerhans cell histiocytosis. Med Pediatr Oncol. (1994) 23:81–5. 10.1002/mpo.2950230204 8202046

[16] Li Y Shi C Wu Y He M Xia X Liu J. Case report: rare systemic and aggressive ALK-Positive histiocytosis with recurrent pancreatitis treating by alectinib. Front Med. (2022) 9:840407. 10.3389/fmed.2022.840407 35665359 PMC9160658

[17] Kemps PG Picarsic J Durham BH Hélias-Rodzewicz Z Hiemcke-Jiwa L van den Bos C. ALK-positive histiocytosis: a new clinicopathologic spectrum highlighting neurologic involvement and responses to ALK inhibition. Blood. (2022) 139:256–80. 10.1182/blood.2021013338 34727172 PMC8759533

[18] Huang H Gheorghe G North PE Suchi M. Expanding the phenotype of ALK-positive histiocytosis: a report of 2 cases. Pediatr Dev Pathol. (2018) 21:449–55. 10.1177/1093526617740784 29224419

[19] Qiu L Weitzman SP Nastoupil LJ Williams MD Medeiros LJ Vega F. Disseminated ALK-positive histiocytosis with KIF5B-ALK fusion in an adult. Leuk Lymphoma. (2021) 62:1234–8. 10.1080/10428194.2020.1861273 33353436

[20] Osako T Kurisaki-Arakawa A Dobashi A Togashi Y Baba S Shiozawa S. Distinct clinicopathologic features and possible pathogenesis of localized ALK-positive histiocytosis of the breast. Am J Surg Pathol. (2022) 46:344–52. 10.1097/PAS.0000000000001794 34482333

[21] Kashima J Yoshida M Jimbo K Izutsu K Ushiku T Yonemori K. ALK-positive histiocytosis of the breast: a clinicopathologic study highlighting spindle cell histology. Am J Surg Pathol. (2021) 45:347–55. 10.1097/PAS.0000000000001567 32826530

[22] Kurita A Yoshida M Murata T Yoshida A Uchiyama N Takayama S. case of ALK-positive histiocytosis with multiple lesions in the unilateral breast: a case report. Int J Surg Case Rep. (2022) 97:107435. 10.1016/j.ijscr.2022.107435 35908452 PMC9403183

[23] Zeng H Liu Y Wang W Tang Y Tian P Li W. rare KIF5B-ALK fusion variant in a lung adenocarcinoma patient who responded to crizotinib and acquired the ALK L1196M mutation after resistance: a case report. Ann Palliat Med. (2021) 10:8352–7. 10.21037/apm-20-2081 33832282

[24] Bai Y Sun W Niu D Yang X Diao X Yu Y. Localized ALK-positive histiocytosis in a Chinese woman: report of a case in the lung with a novel EML4-ALK rearrangement. Virchows Arch. (2021) 479:1079–83. 10.1007/s00428-021-03092-8 33825946

[25] Tran TAN Chang KTE Kuick CH Goh JY Chang CC. Local ALK-Positive Histiocytosis With Unusual Morphology and Novel TRIM33-ALK Gene Fusion. Int J Surg Pathol. (2021) 29:543–9. 10.1177/1066896920976862 33243034

[26] Ezra N Van Dyke GS Binder SW. CD30 positive anaplastic large-cell lymphoma mimicking Langerhans cell histiocytosis. J Cutan Pathol. (2010) 37:787–92. 10.1111/j.1600-0560.2009.01430.x 19817947

[27] Mariño-Enríquez A Dal Cin P ALK. as a paradigm of oncogenic promiscuity: different mechanisms of activation and different fusion partners drive tumors of different lineages. Cancer Genet. (2013) 206:357–73. 10.1016/j.cancergen.2013.07.001 24091028

[28] Stoica GE Kuo A Powers C Bowden ET Sale EB Riegel AT. Midkine binds to anaplastic lymphoma kinase (ALK) and acts as a growth factor for different cell types. J Biol Chem. (2002) 277:35990–8. 10.1074/jbc.M205749200 12122009

[29] Motegi A Fujimoto J Kotani M Sakuraba H Yamamoto T. ALK receptor tyrosine kinase promotes cell growth and neurite outgrowth. J Cell Sci. (2004) 117:3319–29. 10.1242/jcs.01183 15226403

[30] Wu J Savooji J Liu D. Second- and third-generation ALK inhibitors for non-small cell lung cancer. J Hematol Oncol. (2016) 9:19. 10.1186/s13045-016-0251-8 26951079 PMC4782349

[31] Tian Y Li J Liu B Xie H Zheng M Yao W. ALK-positive histiocytosis with disseminated disease responded to alectinib: a case report. Ann Palliat Med. (2021) 10:10095–101. 10.21037/apm-21-2117 34628929

[32] Heigener DF Reck M. Crizotinib. Recent Results Cancer Res. (2018) 211:57–65. 10.1007/978-3-319-91442-8_4 30069759

[33] Shaw AT Bauer TM de Marinis F Felip E Goto Y Liu G. First-line lorlatinib or crizotinib in advanced ALK-positive lung cancer. N Engl J Med. (2020) 383:2018–29. 10.1056/NEJMoa2027187 33207094

[34] Camidge DR Otterson GA Clark JW Ignatius Ou SH Weiss J Ades S. Crizotinib in patients with MET-amplified NSCLC. J Thorac Oncol. (2021) 16:1017–29. 10.1016/j.jtho.2021.02.010 33676017

[35] Alizadeh M Ravindran A Chkheidze R Goyal G Hosseini M Shobeiri P. ALK-positive histiocytosis involving the cavernous sinus: a deceptive radiologic mimic of meningioma. Radiol Case Rep. (2023) 18:2259–63. 10.1016/j.radcr.2023.03.034 37123042 PMC10130913

[36] Wu YL Tsuboi M He J John T Grohe C Majem M. Osimertinib in resected EGFR-mutated non-small-cell lung cancer. N Engl J Med. (2020) 383:1711–23. 10.1056/NEJMoa2027071 32955177

[37] Choi YL Soda M Yamashita Y Ueno T Takashima J Nakajima T. EML4-ALK mutations in lung cancer that confer resistance to ALK inhibitors. N Engl J Med. (2010) 363:1734–9. 10.1056/NEJMoa1007478 20979473

[38] Sasaki T Koivunen J Ogino A Yanagita M Nikiforow S Zheng W. A novel ALK secondary mutation and EGFR signaling cause resistance to ALK kinase inhibitors. Cancer Res. (2011) 71:6051–60. 10.1158/0008-5472.CAN-11-1340 21791641 PMC3278914

[39] Tibaldi C. Mechanisms of resistance to crizotinib in patients with ALK gene rearranged non-small-cell lung cancer. Pharmacogenomics. (2014) 15:133–5. 10.2217/pgs.13.236 24444403

